# Mechanisms of Anthracycline-Induced Cardiotoxicity: Is Mitochondrial Dysfunction the Answer?

**DOI:** 10.3389/fcvm.2020.00035

**Published:** 2020-03-12

**Authors:** Alessandra Murabito, Emilio Hirsch, Alessandra Ghigo

**Affiliations:** Department of Molecular Biotechnology and Health Sciences, Molecular Biotechnology Center, University of Turin, Turin, Italy

**Keywords:** mitochondria, anthracycline, reactive oxygen species, mitochondria-targeted drug, cardiotoxicity after chemotherapy

## Abstract

Cardiac side effects are a major drawback of anticancer therapies, often requiring the use of low and less effective doses or even discontinuation of the drug. Among all the drugs known to cause severe cardiotoxicity are anthracyclines that, though being the oldest chemotherapeutic drugs, are still a mainstay in the treatment of solid and hematological tumors. The recent expansion of the field of Cardio-Oncology, a branch of cardiology dealing with prevention or treatment of heart complications due to cancer treatment, has greatly improved our knowledge of the molecular mechanisms behind anthracycline-induced cardiotoxicity (AIC). Despite excessive generation of reactive oxygen species was originally believed to be the main cause of AIC, recent evidence points to the involvement of a plethora of different mechanisms that, interestingly, mainly converge on deregulation of mitochondrial function. In this review, we will describe how anthracyclines affect cardiac mitochondria and how these organelles contribute to AIC. Furthermore, we will discuss how drugs specifically targeting mitochondrial dysfunction and/or mitochondria-targeted drugs could be therapeutically exploited to treat AIC.

## Introduction

Advances in cancer therapy resulted in marked improvements in patient survival, with anthracyclines (ANTs) probably being the most potent antineoplastic therapeutics available for the clinical practice, and still representing one of the pillars in the treatment of different tumors. In 2018 more than 3 million people were diagnosed with cancer in Europe only, and it has been estimated that currently 14.5 million people are living with a history of cancer in USA, with this number rising up to 19 million over the next 10 years (1, 2). Notably, 50% of people diagnosed with cancer today will survive at least 10 years after diagnosis, and this proportion is even higher for childhood cancer survivors. However, this improvement in survival of cancer patients has led to a greater recognition of the long-term adverse effects of antineoplastic therapies like ANTs, mostly involving the cardiovascular system. In a cohort of almost 2,000 cancer survivors monitored over 7 years, 33% of deaths were related to cardiovascular conditions while cancer-related mortality accounted for 51% of deceases. Given the concrete possibility of incurring in ANT-induced cardiotoxicity (AIC), and that the number of cancer survivors is constantly increasing, in the upcoming years there will probably be a Cardio-Oncology “epidemic.” For this reason, cardiologists, oncologists, and basic scientists are combining their efforts in order to better characterize the molecular mechanisms behind this pathology (3). In this regard, in recent years the role of mitochondria has strongly emerged, since several compounds exert their cardiotoxic effects targeting these organelles (4, 5). This is due to the fact that mitochondria are particularly important for the heart because of its high demand in energy. Since mitochondria are the organelles dedicated to ATP production, dysfunctional mitochondria are repeatedly replaced by newly synthesized ones with the purpose of sustaining the constant need for ATP, underlying the importance of mitochondria dynamics and mitophagy. Drugs that impair the proper activity of mitochondria likely cause a substantial decrease in ATP levels that, eventually, leads to myocardial dysfunction (6). For this reason, drugs preserving mitochondrial function and metabolism are receiving increasing attention in order to treat or prevent cardiotoxicity induced by several drugs, including ANTs. In this review, we will describe the crucial role in AIC of mitochondria, organelles of fundamental importance for the heart, and we will discuss about specific treatments targeting their function and metabolism.

## AIC: From Definition to Current Treatment

ANTs, such as doxorubicin (DOX), daunorubicin and epirubicin, are antibiotic agents highly effective as anticancer therapeutics, and for this reason they have been registered by the World Health Organization as essential medicines (7). However, it was noticed early on that their use is associated to the development of heart failure (HF) (8, 9). Already in the seventies, Von Hoff et al. analyzed retrospectively more than 4,000 DOX-treated subjects and found that the overall incidence of congestive HF caused by the treatment was 2.2%. Notably, the number of patients affected by AIC in this study is probably underestimated since it was based only on clinician-identified signs and symptoms of congestive HF. Moreover, it was already clear that the probability of incurring in AIC is strictly dependent on the total dose administered and that the use of smaller, divided doses of DOX decreases the likelihood of developing cardiotoxicity, while there is a sharp increase in the prevalence of HF occurring at increasing doses of the drug (10). Importantly, anthracyclines are rarely administered as single agents and are more often combined with radiotherapy or modern targeted therapies, like monoclonal antibodies, which importantly exacerbate toxicity (11).

AIC can manifest acutely, early after infusion, strongly compromising cancer treatment since it may require dose modification or even cessation of anticancer therapies (12). Almost 30% of patients are affected by this type of cardiotoxicity, that is characterized by electrocardiogram abnormalities, including atypical ST changes, reduced QRS voltage, tachycardia, and supraventricular premature beats. Yet, acute AIC is a rare complication and the most prevailing and significant form of AIC is the chronic one. It is characterized by left ventricular systolic dysfunction, with a reduction in left ventricular ejection fraction (LVEF), which can be very insidious since it is asymptomatic in the early stages. It can eventually progress to dilated cardiomyopathy and congestive heart failure (CHF), which is nowadays one of the main co-morbidity in childhood cancer survivors (11, 13, 14). These patients have a 12-fold increased chance of developing congestive heart failure (CHF) up to 30 years after treatment, with an occurrence of AIC up to 30% (15–17). Of notice, some cancer patients already have pre-existing cardiovascular diseases or at least cardiovascular risk factors that strongly increase the likelihood of developing cardiac issues, and specifically AIC, in these individuals.

The assessment of AIC primarily relies on evaluation of clinical symptoms and/or detection of systolic function (LVEF) by echocardiography, acquisition scans, and magnetic resonance imaging (18). In particular, cardiotoxicity is currently diagnosed when a decline of 5–55% in LVEF with HF symptoms, or an asymptomatic decline of 10 to below 55%, is observed. Nevertheless, recent studies highlight the limitations of these ejection fraction-based screenings, proposing new diagnostic strategies. In particular, strain rate imaging and troponin (Tn) leakage in the peripheral blood could be used to identify patients with early clinical signs of cardiotoxicity (19–21). From a therapeutic point of view, unfortunately there is no specific treatment targeting AIC. Efforts are being made to develop strategies to prevent AIC that, depending on their mechanism of action, are classified as primary, when focused on preventing the disease concomitantly with ANT treatment, and secondary, when prompted to prevent symptomatic progression (22). For now though all the secondary preventive strategies have limited follow up, also because of the difficulties related to monitoring cardiotoxicity in both adults and children (22). Some clinical trials have shown modest success with the usage of the standard pharmacological regimen for HF. Notably, it has been reported that the non-selective β adrenergic receptor (βAR) blocker, carvedilol, can prevent DOX-induced left ventricular dysfunction through its antioxidant properties, and can ameliorate cardiac function and survival in cancer patients under ANT therapy (23–25). More recently, it was demonstrated that early treatments with the angiotensin converting enzyme I (ACE-I) enalapril, either alone or in combination with carvedilol, are able to fully or partially recover LVEF in 82% of patients manifesting signs of cardiotoxicity within the first year after the end of ANT treatment (13). Unfortunately, these regimens are far from optimal for AIC treatment, and this is probably due to the fact that the mechanisms involved in this specific type of cardiomyopathy are different to those underlying other types of cardiac disease, like ischemic, post-infectious, and idiopathic dilated cardiomyopathies (22). This underlies the need for more specific therapeutics, and so, of a better understanding of the molecular mechanisms behind this condition.

## Mitochondria: Key Players in AIC

If the molecular processes behind the anticancer effects of ANTs are well-known and studied, the mechanisms underlying their cardiotoxic effects are still poorly understood and controversial. It is well-established that ANTs exert their anticancer action by directly targeting and inhibiting topoisomerase 2 (Top2) in cancer cells, more specifically the 2α isoform, halting DNA transcription, and replication (26). However, the same mechanism can hardly explain the toxic effect of ANTs on the heart, since cardiomyocytes are for definition non-dividing cells, thus leaving an open question for cardio-oncology researchers (27, 28). Recent evidence suggests that DOX cardiotoxicity is causally linked to inhibition of a Top2 isoform which is preferentially expressed by differentiated cells, like cardiomyocytes, namely Top2β, the only Top2 expressed in mitochondria (27, 29). Moreover, a number of other mechanisms of AIC, which are not necessarily linked to Top2β inhibition, have started to emerge. Interestingly, both pathways have been reported to impact on the activity of mitochondria. In the next paragraphs, we will describe Top2β-dependent (or direct) and Top2β-independent (indirect) mechanisms of DOX cardiotoxicity and how these signaling pathways converge on the dysregulation of mitochondrial activity and metabolism in cardiomyocytes.

### “Direct” Mechanisms of AIC Involving Mitochondria

**Figure 1:**
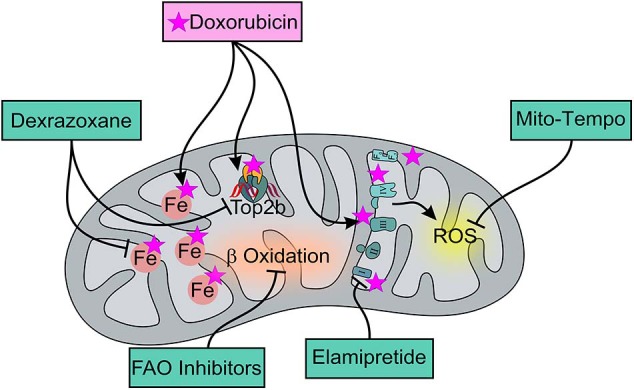
Effects of DOX and of mitochondria-targeted drugs on mitochondrial function and metabolism. DOX preferentially accumulates within mitochondria thanks to its ability to specifically bind to the phospholipid cardiolipin, causing membrane perturbation and ETC disruption that can be limited by Elamipretide, a tetrapeptide that improves the efficiency of electron transport and restores cellular bioenergetics. ETC dysfunction mainly induces ROS production that can be though limited by the usage of the mitochondria-targeted antioxidant, Mito-Tempo, a specific scavenger of mitochondrial superoxide. Moreover, DOX can directly interact with iron to form reactive ANT-iron complexes resulting in an iron cycling between Fe^3+^ and Fe^2+^ which is associated with ROS production and altered iron homeostasis. Dexrazoxane, as an iron-chelator, can inhibit the production of ROS ensuing from the interaction between ANT and non-heme iron, ultimately alleviating DOX-induced mitochondrial oxidative stress. Moreover, Dexrazoxane can prevent DOX from binding to the Top 2β-DNA complex. For AIC treatment, FAO inhibitors can also be used for their ability to enhance glucose oxidation and prevent a decrease in intracellular ATP levels, thereby ensuring the proper maintenance of cellular homeostasis.

As mentioned above, the cellular targets of DOX are topoisomerases, more specifically of the Top2 class (30). DOX can bind both DNA and Top2 in order to form the ternary Top2-DOX-DNA cleavage complex which triggers cell death. As mentioned before, besides inhibiting Top2α in proliferating cells, ANTs can target Top2β, which is also the only known type 2 topoisomerase present in cardiac mitochondria [Figure 1; (27)]. In their study, Zhang et al. demonstrated that DOX treatment induces significant changes in the expression of genes controlling both mitochondrial structure and metabolism (oxidative phosphorylation pathways) in cardiomyocytes expressing Top2β (Top2β^+/+^), but not in Top2β knockout mice (Top2β^Δ/Δ^) (29). More specifically, among the genes downregulated after DOX treatment in Top2β^+/+^, and not significantly affected in Top2β^Δ/Δ^ cardiomyocytes, are Ndufa3 (encoding the NADH dehydrogenase 1-α subcomplex 3), Sdha (encoding succinate dehydrogenase complex II, subunit A), and Atp5a1 (encoding the ATP synthase subunit α). In agreement, mitochondria fail to maintain their membrane potential in DOX-treated Top2β^+/+^ but not in Top2β^Δ/Δ^ cardiomyocytes (29). In addition to modulation of genes involved in mitochondrial function and metabolism, DOX was also shown to decrease the transcription of *Ppargc1a* and *Ppargc1b*. These two genes encode for PGC-1α and PGC-1β, respectively, that by interacting with crucial transcription factors, namely NRF-1, NRF-2, and ERRα, push the expression of genes implicated in mitochondrial biogenesis (29). In keeping with their preserved mitochondrial function, cardiomyocyte-specific Top2β knockout mice are protected from DOX-induced progressive HF. Indeed, after 5 weeks of DOX treatment, Top2β^+/+^ mice show a decrease in ejection fraction up to 50%, whereas this parameter is not altered in Top2β^Δ/Δ^ mice. Zhang et al. also demonstrated that reactive oxygen species (ROS) production is reduced by 70% in the hearts of Top2β^Δ/Δ^ as compared to Top2β^+/+^ mice (29). Of note, the finding that Top2β silencing only partially reduces ROS production in cardiomyocytes treated with ANTs suggests that ROS may be generated in response to DOX by additional Top2β-independent mechanisms that will be discussed in the next paragraph.

### “Indirect” Mechanisms of AIC Involving Mitochondria

Since the initial discovery of ANT cardiotoxicity, the generation of excessive ROS has represented the most widely accepted mechanistic explanation. Even if in cardiomyocytes ROS can be produced, at least in part, as a consequence of ANT-mediated Top2β inhibition (see previous paragraph for further detail), several “indirect” or Top2β-independent mechanisms significantly contribute to ROS production and mitochondrial dysfunction. In the next paragraph, we will describe mechanisms of AIC which are unrelated to Top2β inhibition and that culminate in alterations of mitochondrial function and metabolism.

#### Mitochondrial ROS Production and Metabolism Dysregulation

Recent evidence suggests that ANTs, in particular DOX, preferentially accumulate in the mitochondria of cardiomyocytes, strongly impacting on both the structure and the activity of these organelles. Indeed, DOX can directly bind to the abundant phospholipid cardiolipin, located in the inner mitochondrial membrane (31, 32). This interaction hampers the electron transport chain (ETC), since it inhibits complex I and II, leading to ROS production (Figure 1). More specifically, a quinone moiety in the C ring of DOX can accept electrons for NADH or NADPH and is thus reduced by the respiratory chain complex I, generating a reactive semiquinone free radical (33, 34). On one hand, this mechanism decreases the electron flow through the ETC, removing electrons normally used for ATP production; on the other hand, the reduced semiquinone can transfer the electron to O_2_, generating the superoxide anion ${\text{O}}_{2}^{-}$. DOX can be generated back by this process, in a reaction known as the “redox cycling,” and can be reduced again if NADH is present, producing ${\text{O}}_{2}^{-}$ continuously. ${\text{O}}_{2}^{-}$ can be transformed into the low-toxic hydrogen peroxide (H_2_O_2_) by superoxide dismutase (SOD) or into other ROS (35, 36). ANT-mediated production of these reactive species in turn can activate different pathways leading to cardiomyocytes death, including apoptosis and necrosis. Intriguingly, DOX-induced cardiomyopathy has been recently linked to another form of regulated cell death, the less characterized iron-dependent cell death, also named ferroptosis, which is driven by iron-dependent lipid peroxidation. Indeed, ANTs produce ROS also because they can chelate free iron, leading to the formation of reactive iron-DOX complexes that can interact with O_2_ [Figure 1; (37)]. Moreover, it has been shown that DOX can upregulate heme oxygenase 1, the enzyme responsible for heme degradation, and releases free iron in cardiomyocytes, leading to oxidation of lipids of the mitochondrial membrane and to a further release of free iron in cardiomyocytes, thus feeding this vicious cycle of ROS production (37). In addition, Ichikawa et al. showed that DOX specifically triggers iron accumulation in the mitochondria of isolated cardiomyocytes, without altering total cellular iron levels. Intriguingly, this preferential accumulation is also found in the hearts of DOX-treated patients. Mechanistically, the increase in mitochondrial iron levels upon ANT administration is mediated by the downregulation of the ATP-binding cassette subfamily B member 8 (ABCB8), a transporter protein mediating mitochondrial iron export. ABCB8 overexpression protects mice from DOX-induced oxidative stress and cardiomyopathy and preserves mitochondrial structure and cardiomyocyte viability. Conversely, in the absence of ABCB8, DOX-induced ROS production and mitochondrial damage are increased compared to controls, underlying the cardio-protective role of this transporter (37, 38). Notably, other aspects of mitochondrial metabolism and energy production can be disrupted by ANTs. It has been demonstrated that β-oxidation, the main process used by the healthy heart to generate energy, is inhibited upon DOX treatment through the down-modulation of carnitine palmitoyltransferase 1 (CPT-1), while glycolysis is increased by 50% within few hours as a compensatory response. However, this metabolic adaptation is reversed with time, with a strong decrease in glucose oxidation that has been demonstrated both *in vitro* and *in vivo*. This may be due to the reduction of glucose supply after the induction phase or because of the poor availability of one of the key enzymes of the process, namely phosphofructokinase (PFK) (39).

#### Calcium Homeostasis Dysregulation

The metabolic changes induced by DOX, and the consequent reduction in ATP levels, are known to negatively impact myocardial contractility, which may be exacerbated by an impairment of myocardial Ca^2+^ signaling. It is known that DOX affects Ca^2+^ homeostasis and signaling via several mechanisms, also involving ROS. On one hand, the lipid peroxidation elicited by DOX-mediated ROS production can alter the activity of membrane-residing proteins, such as mitochondrial calcium channels (40, 41). In addition, ANTs can impair the expression and activity of key players of myocardial contraction, namely the cardiac ryanodine receptor (RyR2) and the sarco-/endoplasmic reticulum Ca^2+^ ATPase (SERCA2) (42). In physiological conditions, the action potential mediating contraction is detected by L-type Ca^2+^ channels that activate RyR2, which are responsible for Ca^2+^ release from the sarcoplasmic reticulum (SR). This latter increase in cytoplasmic Ca^2+^ level triggers muscle contraction. Ca^2+^ levels are eventually restored to basal via the activation of SERCA2, mediating the reuptake of Ca^2+^ into the SR (42). DOX and its main metabolite, doxorubicinol (doxOL), are known to activate and increase the open probability of RyR2, though this effect is acute and detectable only right after administration of the drug and at low concentrations (42). Instead, doxOL was found to oxidize RyR2 thiols and this irreversible modification causes a significant inhibition of the channel. Interestingly, it has been shown that SERCA2 can be inhibited via the same oxidation process, which leads to a dramatic increase in cytoplasmic Ca^2+^ levels (42). In addition, this process is exacerbated by the fact that ANTs can negatively affect the transcription of the channel (42). More importantly, DOX is able to activate Calcium/Calmodulin-dependent protein kinase-II (CaMKII), which alters mitochondrial Ca^2+^ homeostasis and promotes apoptosis. CaMKII increases Ca^2+^ influx in mitochondria through mitochondrial calcium Ca^2+^ uniporter (MCU) via activation of the nuclear factor-kappa B (NF-kB) and p53. This, in turn, leads to the opening of the permeability transition pore (MTP) at lower levels of Ca^2+^ compared to normal conditions, resulting in dissipation of the mitochondrial membrane potential and in increased permeability to apoptotic factors (43, 44). Moreover, ANT-mediated ATP depletion (as described in the previous paragraph) also reduces the mitochondrial membrane potential and causes MTP opening, further dysregulating Ca^2+^ homeostasis (45).

#### Autophagy and Mitochondrial Dynamism Impairment

Among all mammalian cells, cardiomyocytes emerge for having the highest mitochondrial density and also the greatest respiratory capacity. This might be the reason why preserving the homeostasis of these organelles is a physiological imperative for the heart. In agreement, mitochondria damaged by DOX have to be promptly removed to maintain a healthy heart. Unfortunately, ANTs are known to disrupt the major degradative/recycling process of mitochondria, namely autophagy (46, 47). Several studies found that acute administration of high-dose ANTs can induce the accumulation of both LC3 and p62, the major autophagy markers, with a reduction in ATP levels in mouse hearts, and a significant suppression of oxygen consumption rate (OCR) in their mitochondria (46). Further analysis from Li et al. demonstrated that DOX blocks cardiomyocytes autophagic flux mediating a strong accumulation of undegraded autolysosomes. This is due to defects in lysosomal acidification caused by DOX-mediated suppression of the activity of V-ATPase, the proton pump that generates and maintains pH gradients in this organelle (48). Furthermore, ANTs inhibit the phosphorylation of one of the positive regulators of autophagy initiation, AMPK, suggesting that ANTs dampen autophagy not only by impairing the autophagic flux but also by inhibiting its initiation. Starvation prior to ANT treatment restores AMPK signaling and autophagy, ultimately protecting the heart against cardiac dysfunction (49). Another mechanism by which DOX impairs autophagy involves the PI3Kγ pathway. Li et al. recently showed that DOX activates a PI3Kγ/Akt/mTOR cascade which ultimately converges on autophagy inhibition, while genetic or pharmacological inhibition of PI3Kγ restores the autophagic flux and protects mice against AIC (50).

Along with impaired autophagy, AIC is characterized by defective mitochondrial dynamics, which refers to organelle fusion, fission, and mitophagy, a specific autophagic mechanism targeting mitochondria. The mitochondrial fusion proteins, mitofusin1 and 2 (Mfn1 and Mfn2), and optic atrophy 1 (Opa1), as well as the mitochondrial fission protein, dynamin related protein (Drp)1, are highly expressed in the mammalian heart, wherein their genetic ablation causes dramatic cardiac dysfunction. Mfn2 levels are decreased in cardiomyocytes after treatment with DOX and this event is associated with increased mitochondrial fission, leading to mitochondrial fragmentation, mitophagy, decreased antioxidative capacity, and ultimately cell death. Accordingly, increased expression of Mfn2 in cardiomyocytes, or the use of the mitochondria-targeted antioxidant Mito-Tempo, a specific scavenger of mitochondrial superoxide, attenuate DOX-induced mitochondrial fission and prevent cardiomyocyte mitochondrial ROS production and apoptosis (51). Mito-Tempo though is not the only known compound to counteract AIC. Several others are now being investigated and will be extensively described in the following paragraphs.

## Targeting Mitochondria and Their Metabolism for the Treatment of AIC

In-depth study of the intertwined molecular mechanisms underlying ANT-induced mitochondrial toxicity has recently paved the way to the development of approaches potentially useful to treat AIC. However, targeting AIC in the clinical setting is still challenging, since a major requirement for these medications is that they do not interfere with the antitumor activity of ANTs. Below we will describe the most promising therapeutics for AIC, with a major focus on those targeting either ROS and their production, or mitochondrial metabolism.

### Dexrazoxane

Dexrazoxane is not only one of the most studied cardio-protective adjuvant for DOX chemotherapy, but it is also the only Food and Drug Administration (FDA)- and European Medicines Agency (EMA)-approved drug for AIC prevention (12, 52). Thanks to its ability to act as an iron-chelator, dexrazoxane inhibits the production of ROS ensuing from the interaction between ANTs and non-heme iron, ultimately alleviating DOX-induced mitochondrial oxidative stress [Figure 1; (53, 54)]. However, the concept that dexrazoxane promotes cardioprotection only by virtue of its antioxidant properties is debated, especially in view of the finding that other antioxidant drugs, such as vitamin A, vitamin E, and N-acetylcysteine, failed to provide benefits in the treatment of AIC (55–57). An additional mechanism that may account for the cardioprotective action of dexrazoxane is its ability to prevent DOX from binding to the Top2β-DNA complex. X-ray crystal structure analyses revealed that dexrazoxane can bind to the two ATP binding sites at the N terminus of Top2 and bridges two Top2 monomers in the closed-clamp configuration [Figure 1; (58)]. Moreover, it has also been demonstrated that dexrazoxane forms a tight complex with the ATPase domain of human Top2α and Top2β, suggesting that this compound prevents ANT from binding to Top2 (59). In addition, dexrazoxane has been shown to interact with Poly(ADP-ribose) (PAR) monomers, acting as a PAR Poly(ADP-ribose) polymerase (PARP) inhibitor (60). In agreement, inhibition of this enzyme improves cardiac function and decreases mortality without altering the anticancer activity of DOX in several animal models of DOX-induced cardiomyopathy (61). Consistent with its mechanisms of action, dexrazoxane is exploited to prevent rather than treat AIC and its use appears to be most appropriate in patients with stage A of HF, i.e., at high risk of developing the pathology. However, Ganatra et al. demonstrated that dexrazoxane exerts its cardioprotective function also in stage B HF (62). In a small cohort of patients showing pre-existing asymptomatic, systolic left ventricular (LV) dysfunction, the administration of dexrazoxane 30 min before each ANT dose was enough to allow patients to complete their planned chemotherapy, with a minimal decrease in LVEF and no elevation in HF biomarkers. On the contrary, the three patients that did not receive dexrazoxane had a marked reduction in heart function and developed HF. Of note, two of them died from cardiogenic shock and multi-organ failure (62).

Concerning the clinical efficacy of dexrazoxane, it has been shown in multiple trials that it can reduce the incidence of CHF and LVEF decline in patients treated with ANTs (63–65). These findings were also corroborated by a more recent study in which Marty et al. found that, based on both LVEF and CHF results, 164 relapsed breast cancer patients treated with dexrazoxane have significantly lower overall cardiac events in comparison with the control group treated with DOX or epirubicin only (66). Similarly, dexrazoxane has been shown to abrogate DOX-mediated mitochondrial dysfunction in childhood cancer survivors. Lipshultz et al. found that, in peripheral blood mononuclear cells (PBMCs), DOX-damaged mitochondria expand their mtDNA, which encodes for 13 polypeptides involved in oxidative phosphorylation, as an attempt to compensate for the injury and improve mitochondrial metabolism (67). Treatment with dexrazoxane, together with DOX, reduces the number of mtDNA copies per cell compared to the group treated with DOX only, suggesting preserved mitochondrial function in patients receiving the combination therapy (67). Intriguingly, besides proving the efficacy of dexrazoxane in counteracting AIC-related mitochondrial dysfunction, this study also suggests that mitochondrial injury, and the ensuing increase of mtDNA in peripheral blood, might represent a biomarker for early detection of cardiotoxicity, which still represents an unmet clinical need.

Despite evident clinical benefits, in 2011 EMA contraindicated the usage of dexrazoxane in children since its efficacy in this sub-population was not assessed. In addition, it was proposed that dexrazoxane could not only attenuate the anticancer effects of ANTs and increase the risk of secondary malignancies, but could also cause myelotoxicity (64–66). Nevertheless, this view has been recently refuted by a number of studies (68). A phase-III clinical trial, involving more than 500 children and adolescents affected by T-cell acute lymphoblastic leukemia (ALL) or lymphoblastic non-Hodgkin lymphoma, was conducted to investigate not only the cardio-protective effects of dexrazoxane but also its safety as well as its potential impact on the antineoplastic efficacy of ANTs (69). In addition, Lipshultz et al. found that dexrazoxane attenuates DOX-induced cardiac injury in children with acute lymphoblastic leukemia, without compromising its antileukemic efficacy (70). It was also reported that dexrazoxane alone does not increase the risk of second primary malignancies (SPMs), which are instead related to the usage of three Top2 inhibitors used in combination (doxorubicin, etoposide, and dexrazoxane) and mostly etoposide (71). For these reasons, EMA has approved the administration of dexrazoxane to children supposed to be given more than 300 mg/m^2^ of ANTs (12, 52, 68).

### Mito-Tempo

The novel drug named mitochondrial-targeted Tempo l (Mito-Tempo) is a well-known superoxide dismutase (SOD) mimetic. Mitochondria are the only organelles having a unique type of superoxide dismutase, the manganese-containing SOD2, which is crucial for protecting against excessive production of O_2_^−^, a key feature of AIC (Figure 1). Mice that do not express this protein develop a severe cardiomyopathy already at 10 days after birth, while mice missing one allele of SOD2 (SOD2^+/−^ mice) develop hypertension with time and if challenged with an high-salt diet, suggesting a role for this enzyme in cardiac protection (72). Mito-Tempo consists of the tempol moiety bound to a triphenylphosphonium cation that allows the molecule to enter mitochondria, and this is the reason why this molecule may be highly effective in organs, such as the heart, which are rich in these organelles. Mimicking the activity of SOD, Mito-Tempo acts as an antioxidant drug in rats, and in mice it has also been shown to alleviate oxidative stress and cardiac toxicity induced by DOX (73, 74). Indeed, already in the 90's, it was demonstrated that Mito-Tempo significantly reduces the contractile impairment as well as the lipid peroxidation observed in rat heart treated acutely with DOX (75). In all these *in vivo* studies, Mito-Tempo was used in combination with ANTs in patients with no pre-existing heart disease, suggesting that it might be exploited to prevent AIC likely in patients in stage A HF. In addition, in a guinea pig model of non-ischemic HF, Mito-Tempo reversed the pathological phenotype, suggesting that this compound can also have a therapeutic effect in patients in later stages of ANT-induced HF (76). More recently, Mito-Tempo was used in combination with dexrazoxane and this combinatorial treatment ameliorates DOX-induced cardiomyopathy without altering the antitumor activity of DOX (77).

### Elamipretide

Elamipretide is one of the first drugs developed to target selectively the mitochondrial ETC in order to improve the efficiency of electron transport and restore cellular bioenergetics [Figure 1; (78)]. More than one mechanism of action has been proposed for this tetrapeptide. It penetrates cell membranes, localizing to the inner mitochondrial membrane where it can interact with the phospholipid cardiolipin. Cardiolipin has a crucial role in maintaining the functional positioning of the ETC complexes and supercomplexes within the inner mitochondrial membrane, allowing for efficient electron transfer down the redox chain, minimizing reactive oxygen species production. This binding between cardiolipin and the tetrapeptide prevents peroxidation of the phospholipid, thereby maintaining membrane fluidity and supercomplex formation and enhancing electron transport chain function, ultimately increasing ATP synthesis and reducing mitochondrial ROS (79–82). Several studies conducted in rats showed that elamipretide can significantly improve myocardial mitochondrial ATP content, reduce myocardial infarct size and improve cardiac function (83–85). Moreover, treatment with elamipretide improves left ventricular function in animals with HF (84). Saba et al. also demonstrated a significant improvement in ejection fraction in dogs with HF treated with elamipretide for 3 months (86). In addition, this compound can ameliorate left ventricular relaxation via restoration of cardiac myosin binding protein-C (84, 86, 87). A clinical trial of elamipretide in patients with heart failure with reduced ejection fraction (HFrEF) has also been conducted to evaluate safety, efficacy, and tolerability of the compound. Daubert et al. reported that no subjects suffered any serious adverse events, and only one stopped the treatment after a single administration. Moreover, all patients had stable hemodynamic parameters of blood pressure and heart function, suggesting that elamipretide is well-tolerated also together with current standard HF medications. Most notably, patients treated with elamipretide showed a significant reduction in left ventricular volumes in comparison with placebo, despite the small sample size of the trial (88). Of course, larger studies are required to determine its safety as well as its efficacy in patients with HF, but up to now elamipretide seems to be an optimal therapeutic option for targeting mitochondrial dysfunction in the future. On note, elamipretide has not yet been tested in a specific model of AIC but all these studies suggest that this molecule can both ameliorate and prevent different aspects of mitochondrial dysfunction, leading to envisage its use in patients at different stages of the disease. Unfortunately, there is still no evidence that this drug does not alter the antineoplastic activity of ANTs, which might be a possibility because of its known ability to inhibit apoptosis (84). Further studies are needed to prove the possibility of using this molecule in Cardio-Oncology.

### Autophagy-Targeting Drugs

Until now, no compounds targeting autophagy have been used in clinical trials to prevent AIC or any cardiac disease. Targeting autophagy in AIC, as well as in any disease context, is still controversial, since this process is critical to the maintenance of cellular homeostasis and it has to be finely tuned, with any perturbation being either beneficial or detrimental (47, 89). Some attempts to modulate this process have been reported in animal models and have shown promising starting results, suggesting that inhibiting this process can be protective and that can be used in the future in patients in stage A of AIC. Sciarretta et al. also demonstrated that the autophagy activator trehalose can protect from myocardial infarction-induced cardiac remodeling, suggesting the possible use of this molecule as a therapeutic agent for HF (90). Sishi et al. showed that rapamycin, a known potent activator of autophagy, is able to improve the negative effects mediated by DOX treatment when administered in combination with the anticancer therapy, leading to a decrease in ROS production, and enhanced mitochondrial function (91). Pharmacological inhibition of PI3Kγ phenocopies mTOR blockade and restores the autophagic flux, ultimately preventing AIC (50). However, boosting the autophagic process can negatively impact on the efficacy of cancer treatments since it may make the tumor resistant to chemotherapy. In agreement, autophagy inhibitors, instead of activators, have been tested in oncology so far. Several trials have been carried out inhibiting autophagy with hydroxychloroquine (HCQ), the only clinically-approved autophagy inhibitor (92), raising some concerns about the possible future usage of autophagy-activators for curing AIC.

### Inhibitors of Mitochondrial Fatty Acid Beta Oxidation

Members of this category are Trimetazidine, Ranolazine, and Perhexiline and their use results in the reduction of myocardial fatty acid (FA) uptake and oxidation (Figure 1). In pathological conditions, such as HF, cardiac fatty acid and glucose metabolism are altered and contribute to impaired heart efficiency and function. More specifically, there is an increase in the amount of fatty acids that are oxidized by cardiac mitochondria (93–95). Since FA oxidation (FAO) consumes more energy in comparison with glucose oxidation, requiring 10% more oxygen for a given amount of ATP that is produced, an increase in the amount of FA oxidized by the mitochondria can potentially reduce cardiac efficiency and impair heart function (96). Therefore, FAO inhibitors might represent promising drugs for treating AIC in patients at more advanced stages of the disease, such as B and C, since they lead to an enhanced glucose oxidation and prevent a decrease in intracellular ATP levels, thereby ensuring the proper functioning of ionic pumps and maintenance of cellular homeostasis (97–99). Nevertheless, early and sustained inhibition of CPT-1, the crucial and limiting enzyme of FAO, was shown to prevent LV dysfunction and remodeling, as well as efficiently slowing down the development and progression of the disease, in a dog model of HF, suggesting the possible usage of FAO inhibitors also in stage A HF (100). Of note, these compounds could also provide the opportunity to target cancerous cells as well, since they depend on FAO for several aspects such as proliferation, survival and drug resistance (101).

*Trimetazidine* is an antiischemic agent able to specifically inhibit the long-chain mitochondrial 3-ketoacyl coenzyme A thiolase enzyme that can help cardiomyocytes to maintain proper energy metabolism. No clinical trial has been conducted using this drug for the treatment of AIC, or more generally HF, but its safety and tolerability have been proven through its use in acute coronary syndrome (102). Several studies demonstrated that trimetazidine is effective in improving LVEF, decreasing the rate of hospitalization and reducing brain natriuretic peptide (BNP) levels in subjects with HF (103–106). Moreover, it can also improve cardiac function and reduce HF symptoms when administered together with metoprolol, a βAR blocker.

*Ranolazine*, if used at high concentrations, is a partial inhibitor of fatty acid beta-oxidation (107). Its main mechanism of action is indeed related to its capability to inhibit late inward sodium channels. In failing myocytes, these channels are hyperactivated, leading to calcium overload and in turn contractile dysfunction and increased oxygen consumption (108). Ranolazine is approved for the treatment of chronic angina, but there is evidence suggesting its clinical effect also for HF treatment (109). Up to now, it has been demonstrated that ranolazine mediates diastolic benefits, by restoring myocyte relaxation, reducing resting tension as well as left ventricular end diastolic pressure in animal studies conducted in dogs (110, 111). Further improvements have also been reported when this drug is used in combination with βAR blockers (112). Concerning clinical trials, a small sample size study has been conducted in HF patients with preserved ejection fraction, revealing that ranolazine can provide improvement in hemodynamics, but no evidence was provided of improvement in relaxation parameters (113).

*Perhexiline* is another drug acting on metabolism that was originally thought as an antianginal medication and its usage was declined for several side effects, including hepatotoxicity and neurotoxicity (114). More recently, its toxicity has been found to be preventable with individualized dosing, but its clinical use remains difficult. Its activity as a fatty acid beta-oxidation inhibitor was demonstrated on rat hearts that showed a reduction of fatty acid utilization of 35%, with a concurrent increase in cardiac output of 80 mL/min/g. More specifically, it was demonstrated that perhexiline can inhibit CPT-1, known to control access of long chain fatty acids to the mitochondrial site of beta-oxidation (115). Concerning its clinical use for HF treatment, a small sample size clinical trial has been performed, particularly focused on studying its effect on oxygen consumption. A clear improvement in peak oxygen consumption was found following perhexiline treatment compared to no change in patients treated with a placebo, and improved ejection fraction was also observed, suggesting its possible and effective future employment also for AIC (116).

## Beyond Cardiomyocytes

An important aspect to consider from a therapeutic perspective is that, although the majority of the studies in the field of Cardio-Oncology have focused their attention on the effects of ANTs on cardiomyocytes, these are not the unique cellular population found in the heart. The emerging view is that anticancer compounds also target cardiac fibroblasts and endothelial cells. It has been shown that, both *in vitro* and *in vivo*, DOX affects the differentiation of fibroblasts into myofibroblasts which in turn produce a huge amount of extracellular matrix components, leading to cardiac fibrosis. This process is driven by DOX-dependent ROS that activate TGF-β, the main responsible for fibroblast differentiation (117, 118). Moreover, DOX also modulates the activity of ATM, a kinase which is activated in response to DNA damage induced by oxidative stress. Interestingly, this activation occurs only in cardiac fibroblasts and not in cardiomyocytes, suggesting that this may be a cell-type specific mechanism contributing to AIC (119). How ANTs affect mitochondria in fibroblasts is still unexplored and requires additional work. Instead, more information is available on the role of these organelles in cardiac endothelial cells. Apart from increasing cell permeability and leading to edema formation, DOX can also reduce ATP levels and, in turn, mitochondrial function in these cells (120). Moreover, by means of its interaction with the nitric oxide (NO) synthase, DOX can also interfere with NO production that is essential for endothelial homeostasis (121). However, further studies are needed to further explore the role of these other cardiac cell populations in AIC, hopefully paving the way to the development of new therapeutic options.

## Future Perspectives

Besides the urgent need for new effective therapeutic approaches, another still unresolved issue in the field of Cardio-Oncology is how to predict who is likely to develop cardiotoxicity. Anthracycline dose, patient's age and pre-existent cardiovascular disease only partially explain the interindividual susceptibility to AIC and the prevailing hypothesis is that the sensitivity to anthracyclines has a genetic basis (122). Unveiling the genetic variants that contribute to AIC is of upmost importance since it may give the clinicians the opportunity to identify patients at risk prior the treatment, and potentially modify the therapeutic regimens by using alternative drugs or cardioprotective agents. Early candidate gene association studies (CGAS) and genome-wide association studies (GWAS) have started to reveal the first genes, that are primarily related to drug metabolism and transport, iron metabolism, DNA repair, oxidative stress, and calcium homeostasis, with no genes being directly linked to mitochondrial function regulation (123, 124). However, given the small sample sizes of these studies, additional work is warranted to conclusively validate these variants and to discover new genes implicated in AIC susceptibility. In this scenario, human-induced pluripotent stem cells (hiPSCs) represent an emerging powerful tool since they can be obtained non-invasively from blood samples, can be renewed *in vitro* and are genetically identical to the patients from whom they are derived making them the ideal experimental model for pharmacogenomics research. By exploiting hiPSCs, Knowles et al. recently discovered a number of new genetic variants which also include some genes involved in mitochondrial function regulation (125). In addition, being able to faithfully recapitulate *in vitro* the inter-individual susceptibility to AIC (126), hiPSCs offer the unique opportunity to verify *in vitro*, before the drug is administered to the patient, that the treatment does not cause toxicity, paving the way toward a personalized medicine approach in the field of Cardio-Oncology (123).

## Conclusions

It is now well accepted that mitochondrial dysfunction underlies a broad spectrum of pathologies, ranging from cancer to neurodegenerative and cardiovascular disease. It is not surprising that mitochondria play a key role also in the pathogenesis of AIC, considering the ability of ANTs to bind a phospholipid of the inner mitochondrial membrane, cardiolipin, and thus to accumulate within mitochondria. A number of drugs specifically targeting mitochondrial pathways which are deregulated in pathology as well as a new class of mitochondria-targeted compounds have been developed. While most of them have already been tested in preclinical models of HF, little is still known about their therapeutic potential in the treatment of AIC. Further studies in the appropriate preclinical murine and human models of AIC are awaited to fill this gap.

## Author Contributions

AM and AG wrote the manuscript in consultation with EH.

### Conflict of Interest

AG and EH are co-founders and board members of Kither Biotech, a startup biotech focused on the development of PI3K inhibitors. The remaining author declares that the research was conducted in the absence of any commercial or financial relationships that could be construed as a potential conflict of interest.

## References

[1] European Commission ECIS - European Cancer Information System (2019). Available online at: https://ecis.jrc.ec.europa.eu/ (accessed September 01, 2019).

[2] Siegel RL Miller KD Jemal A Cancer statistics, 2016. CA Cancer J Clin. (2016) 66:7–30. 10.3322/caac.21332 26742998

[3] Zamorano JL Lancellotti P Rodriguez Munoz D Aboyans V Asteggiano R Galderisi M. 2016 ESC position paper on cancer treatments and cardiovascular toxicity developed under the auspices of the ESC committee for practice guidelines: the task force for cancer treatments and cardiovascular toxicity of the European Society of Cardiology (ESC). Eur Heart J. (2016) 37:2768–2801. 10.1093/eurheartj/ehw211 27567406

[4] Waseem M Parvez S. Mitochondrial dysfunction mediated cisplatin induced toxicity: modulatory role of curcumin. Food Chem Toxicol. (2013) 53:334–42. 10.1016/j.fct.2012.11.055 23246825

[5] Varga ZV Ferdinandy P Liaudet L Pacher P. Drug-induced mitochondrial dysfunction and cardiotoxicity. Am J Physiol Heart Circ Physiol. (2015) 309:H1453–67. 10.1152/ajpheart.00554.2015 26386112 PMC4666974

[6] Siasos G Tsigkou V Kosmopoulos M Theodosiadis D Simantiris S Tagkou NM. Mitochondria and cardiovascular diseases-from pathophysiology to treatment. Ann Transl Med. (2018) 6:256. 10.21037/atm.2018.06.21 30069458 PMC6046286

[7] WHO Model Lists of Essential Medicines (2019). Available online at: https://apps.who.int/iris/bitstream/handle/10665/325771/WHO-MVP-EMP-IAU-2019.06-eng.pdf (accessed September 01, 2019).

[8] Lefrak EA Pitha J Rosenheim S Gottlieb JA. A clinicopathologic analysis of adriamycin cardiotoxicity. Cancer. (1973) 32:302–14. 10.1002/1097-0142(197308)32:2<302::AID-CNCR2820320205>3.0.CO;2-2 4353012

[9] Von Hoff DD Layard MW Basa P Davis HL Jr Von Hoff AL Rozencweig M. Risk factors for doxorubicin-induced congestive heart failure. Ann Intern Med. (1979) 91:710–7. 10.7326/0003-4819-91-5-710 496103

[10] Buzdar AU Marcus C Smith TL Blumenschein GR. Early and delayed clinical cardiotoxicity of doxorubicin. Cancer. (1985) 55:2761–5. 10.1002/1097-0142(19850615)55:12<2761::AID-CNCR2820551206>3.0.CO;2-P 3922612

[11] Rochette L Guenancia C Gudjoncik A Hachet O Zeller M Cottin Y. Anthracyclines/trastuzumab: new aspects of cardiotoxicity and molecular mechanisms. Trends Pharmacol Sci. (2015) 36:326–48. 10.1016/j.tips.2015.03.005 25895646

[12] Cai F Luis MAF Lin X Wang M Cai L Cen C. Anthracycline-induced cardiotoxicity in the chemotherapy treatment of breast cancer: preventive strategies and treatment. Mol Clin Oncol. (2019) 11:15–23. 10.3892/mco.2019.1854 31289672 PMC6535635

[13] Cardinale D Colombo A Bacchiani G Tedeschi I Meroni CA Veglia F. Early detection of anthracycline cardiotoxicity and improvement with heart failure therapy. Circulation. (2015) 131:1981–8. 10.1161/CIRCULATIONAHA.114.013777 25948538

[14] Bhakta N Liu Q Ness KK Baassiri M Eissa H Yeo F. The cumulative burden of surviving childhood cancer: an initial report from the St Jude Lifetime Cohort Study (SJLIFE). Lancet. (2017) 390:2569–582. 10.1016/S0140-6736(17)31610-0 28890157 PMC5798235

[15] Armstrong GT Ross JD. Late cardiotoxicity in aging adult survivors of childhood cancer. Prog Pediatr Cardiol. (2014) 36:19–26. 10.1016/j.ppedcard.2014.09.003 26412958 PMC4580976

[16] Boyd A Stoodley P Richards D Hui R Harnett P Vo K. Anthracyclines induce early changes in left ventricular systolic and diastolic function: a single centre study. PLoS ONE. (2017) 12:e0175544. 10.1371/journal.pone.0175544 28407011 PMC5391073

[17] Hilfiker-Kleiner D Ardehali H Fischmeister R Burridge P Hirsch E Lyon AR. Late onset heart failure after childhood chemotherapy. Eur Heart J. (2019) 40:798–800. 10.1093/eurheartj/ehz046 30753420 PMC6821336

[18] Vejpongsa P Yeh ET. Prevention of anthracycline-induced cardiotoxicity: challenges and opportunities. J Am Coll Cardiol. (2014) 64:938–45. 10.1016/j.jacc.2014.06.1167 25169180

[19] Armstrong GT Joshi VM Ness KK Marwick TH Zhang N Srivastava D. Comprehensive echocardiographic detection of treatment-related cardiac dysfunction in adult survivors of childhood cancer: results from the St. Jude Lifetime Cohort Study. J Am Coll Cardiol. (2015) 65:2511–22. 10.1016/j.jacc.2015.04.013 26065990 PMC4539123

[20] Sawaya H Sebag IA Plana JC Januzzi JL Ky B Cohen V. Early detection and prediction of cardiotoxicity in chemotherapy-treated patients. Am J Cardiol. (2011) 107:1375–80. 10.1016/j.amjcard.2011.01.006 21371685 PMC3703314

[21] Cardinale D Sandri MT Martinoni A Tricca A Civelli M Lamantia G. Left ventricular dysfunction predicted by early troponin I release after high-dose chemotherapy. J Am Coll Cardiol. (2000) 36:517–22. 10.1016/S0735-1097(00)00748-8 10933366

[22] Bansal N Adams MJ Ganatra S Colan SD Aggarwal S Steiner R Strategies to prevent anthracycline-induced cardiotoxicity in cancer survivors. Cardio-Oncol. (2019) 5:18 10.1186/s40959-019-0054-5 PMC7048046 32154024

[23] Oliveira PJ Bjork JA Santos MS Leino RL Froberg MK Moreno AJ. Carvedilol-mediated antioxidant protection against doxorubicin-induced cardiac mitochondrial toxicity. Toxicol Appl Pharmacol. (2004) 200:159–68. 10.1016/j.taap.2004.04.005 15476868

[24] Spallarossa P Garibaldi S Altieri P Fabbi P Manca V Nasti S. Carvedilol prevents doxorubicin-induced free radical release and apoptosis in cardiomyocytes *in vitro*. J Mol Cell Cardiol. (2004) 37:837–46. 10.1016/j.yjmcc.2004.05.024 15380674

[25] Kalay N Basar E Ozdogru I Er O Cetinkaya Y Dogan A. Protective effects of carvedilol against anthracycline-induced cardiomyopathy. J Am Coll Cardiol. (2006) 48:2258–62. 10.1016/j.jacc.2006.07.052 17161256

[26] Bodley A Liu LF Israel M Seshadri R Koseki Y Giuliani FC. DNA topoisomerase II-mediated interaction of doxorubicin and daunorubicin congeners with DNA. Cancer Res. (1989) 49:5969–78. 2551497

[27] Capranico G Tinelli S Austin CA Fisher ML Zunino F. Different patterns of gene expression of topoisomerase II isoforms in differentiated tissues during murine development. Biochim Biophys Acta. (1992) 1132:43–8. 10.1016/0167-4781(92)90050-A 1380833

[28] Lyu YL Lin CP Azarova AM Cai L Wang JC Liu LF. Role of topoisomerase IIbeta in the expression of developmentally regulated genes. Mol Cell Biol. (2006) 26:7929–41. 10.1128/MCB.00617-06 16923961 PMC1636731

[29] Zhang S Liu X Bawa-Khalfe T Lu LS Lyu YL Liu LF. Identification of the molecular basis of doxorubicin-induced cardiotoxicity. Nat Med. (2012) 18:1639–42. 10.1038/nm.2919 23104132

[30] Tewey KM Rowe TC Yang L Halligan BD Liu LF. Adriamycin-induced DNA damage mediated by mammalian DNA topoisomerase II. Science. (1984) 226:466–8. 10.1126/science.6093249 6093249

[31] Sarvazyan N. Visualization of doxorubicin-induced oxidative stress in isolated cardiac myocytes. Am J Physiol. (1996) 271:H2079–85. 10.1152/ajpheart.1996.271.5.H2079 8945928

[32] Goormaghtigh E Huart P Praet M Brasseur R Ruysschaert JM. Structure of the adriamycin-cardiolipin complex. role in mitochondrial toxicity. Biophys Chem. (1990) 35:247–57. 10.1016/0301-4622(90)80012-V 2204444

[33] Davies KJ Doroshow JH Redox cycling of anthracyclines by cardiac mitochondria. II. anthracycline radical formation by NADH dehydrogenase. J Biol Chem. (1986) 261:3060–7. 3456345

[34] Chen Y Jungsuwadee P Vore M Butterfield DA St Clair DK. Collateral damage in cancer chemotherapy: oxidative stress in nontargeted tissues. Mol Interv. (2007) 7:147–56. 10.1124/mi.7.3.6 17609521

[35] Tokarska-Schlattner M Zaugg M Zuppinger C Wallimann T Schlattner U. New insights into doxorubicin-induced cardiotoxicity: the critical role of cellular energetics. J Mol Cell Cardiol. (2006) 41:389–405. 10.1016/j.yjmcc.2006.06.009 16879835

[36] Marcillat O Zhang Y Davies KJ. Oxidative and non-oxidative mechanisms in the inactivation of cardiac mitochondrial electron transport chain components by doxorubicin. Biochem J. (1989) 259:181–9. 10.1042/bj2590181 2719642 PMC1138489

[37] Fang X Wang H Han D Xie E Yang X Wei J. Ferroptosis as a target for protection against cardiomyopathy. Proc Natl Acad Sci USA. (2019) 116:2672–680. 10.1073/pnas.1821022116 30692261 PMC6377499

[38] Ichikawa Y Ghanefar M Bayeva M Wu R Khechaduri A Naga Prasad SV. Cardiotoxicity of doxorubicin is mediated through mitochondrial iron accumulation. J Clin Invest. (2014) 124:617–30. 10.1172/JCI72931 24382354 PMC3904631

[39] Tokarska-Schlattner M Wallimann T Schlattner U. Alterations in myocardial energy metabolism induced by the anti-cancer drug doxorubicin. C R Biol. (2006) 329:657–8. 10.1016/j.crvi.2005.08.007 16945832

[40] Mylonas C Kouretas D. Lipid peroxidation and tissue damage. In Vivo. (1999) 13:295–309. 10459507

[41] Angsutararux P Luanpitpong S Issaragrisil S. Chemotherapy-induced cardiotoxicity: overview of the roles of oxidative stress. Oxid Med Cell Longev. (2015) 2015:795602. 10.1155/2015/795602 26491536 PMC4602327

[42] Hanna AD Lam A Tham S Dulhunty AF Beard NA. Adverse effects of doxorubicin and its metabolic product on cardiac RyR2 and SERCA2A. Mol Pharmacol. (2014) 86:438–49. 10.1124/mol.114.093849 25106424 PMC4164980

[43] Dhingra R Guberman M Rabinovich-Nikitin I Gerstein J Margulets V Gang H. Impaired NF-κB signalling underlies cyclophilin D-mediated mitochondrial permeability transition pore opening in doxorubicin cardiomyopathy. Cardiovasc Res. (2019). 10.1093/cvr/cvz240. [Epub ahead of print]. 31566215 PMC7177490

[44] Zhang QL Yang JJ Zhang HS. Carvedilol (CAR) combined with carnosic acid (CAA) attenuates doxorubicin-induced cardiotoxicity by suppressing excessive oxidative stress, inflammation, apoptosis and autophagy. Biomed Pharmacother. (2019) 109:71–83. 10.1016/j.biopha.2018.07.037 30396094

[45] Bai P Canto C Oudart H Brunyanszki A Cen Y Thomas C. PARP-1 inhibition increases mitochondrial metabolism through SIRT1 activation. Cell Metab. (2011) 13:461–8. 10.1016/j.cmet.2011.03.004 21459330 PMC3086520

[46] Abdullah CS Alam S Aishwarya R Miriyala SM Bhuiyan AN Panchatcharam M. Doxorubicin-induced cardiomyopathy associated with inhibition of autophagic degradation process and defects in mitochondrial respiration. Sci Rep. (2019) 9:2002. 10.1038/s41598-018-37862-3 30765730 PMC6376057

[47] Li M Russo M Pirozzi F Tocchetti CG Ghigo A. Autophagy and cancer therapy cardiotoxicity: from molecular mechanisms to therapeutic opportunities. Biochim Biophys Acta Mol Cell Res. (2019) 1867:118493. 10.1016/j.bbamcr.2019.06.007 31233802

[48] Li DL Wang ZV Ding G Tan W Luo X Criollo A. Doxorubicin blocks cardiomyocyte autophagic flux by inhibiting lysosome acidification. Circulation. (2016) 133:1668–87. 10.1161/CIRCULATIONAHA.115.017443 26984939 PMC4856587

[49] Kawaguchi T Takemura G Kanamori H Takeyama T Watanabe T Morishita K. Prior starvation mitigates acute doxorubicin cardiotoxicity through restoration of autophagy in affected cardiomyocytes. Cardiovasc Res. (2012) 96:456–65. 10.1093/cvr/cvs282 22952253

[50] Li M Sala V De Santis MC Cimino J Cappello P Pianca N. Phosphoinositide 3-Kinase gamma inhibition protects from anthracycline cardiotoxicity and reduces tumor growth. Circulation. (2018) 138:696–711. 10.1161/CIRCULATIONAHA.117.030352 29348263

[51] Tang H Tao A Song J Liu Q Wang H Rui T. Doxorubicin-induced cardiomyocyte apoptosis: role of mitofusin 2. Int J Biochem Cell Biol. (2017) 88:55–9. 10.1016/j.biocel.2017.05.006 28483668

[52] European Commission Pharmaceuticals Community Register. Cardioxane Art 13 (2020). Available online at: http://ec.europa.eu/health/documents/community-register/html/ho26321.htm (accessed January 03, 2020).

[53] Kwok JC Richardson DR. The cardioprotective effect of the iron chelator dexrazoxane (ICRF-187) on anthracycline-mediated cardiotoxicity. Redox Rep. (2000) 5:317–24. 10.1179/135100000101535898 11140743

[54] Buss JL Hasinoff BB. The one-ring open hydrolysis product intermediates of the cardioprotective agent ICRF-187 (dexrazoxane) displace iron from iron-anthracycline complexes. Agents Actions. (1993) 40:86–95. 10.1007/BF01976756 8147274

[55] Lipshultz SE Cochran TR Franco VI Miller TL. Treatment-related cardiotoxicity in survivors of childhood cancer. Nat Rev Clin Oncol. (2013) 10:697–710. 10.1038/nrclinonc.2013.195 24165948

[56] Legha SS Wang YM Mackay B Ewer M Hortobagyi GN Benjamin RS. Clinical and pharmacologic investigation of the effects of alpha-tocopherol on adriamycin cardiotoxicity. Ann N Y Acad Sci. (1982) 393:411–8. 10.1111/j.1749-6632.1982.tb31279.x 6959564

[57] Myers C Bonow R Palmeri S Jenkins J Corden B Locker G. A randomized controlled trial assessing the prevention of doxorubicin cardiomyopathy by N-acetylcysteine. Semin Oncol. (1983) 10:53–5. 6340204

[58] Classen S Olland S Berger JM. Structure of the topoisomerase II ATPase region and its mechanism of inhibition by the chemotherapeutic agent ICRF-187. Proc Natl Acad Sci USA. (2003) 100:10629–34. 10.1073/pnas.1832879100 12963818 PMC196855

[59] Roca J Ishida R Berger JM Andoh T Wang JC. Antitumor bisdioxopiperazines inhibit yeast DNA topoisomerase II by trapping the enzyme in the form of a closed protein clamp. Proc Natl Acad Sci USA. (1994) 91:1781–5. 10.1073/pnas.91.5.1781 8127881 PMC43247

[60] McCormack K The cardioprotective effect of dexrazoxane (Cardioxane) is consistent with sequestration of poly(ADP-ribose) by self-assembly and not depletion of topoisomerase 2B. Ecancermedicalscience. (2018) 12:889 10.3332/ecancer.2018.889 30792806 PMC6351063

[61] Pacher P Liaudet L Mabley JG Cziraki A Hasko G Szabo C. Beneficial effects of a novel ultrapotent poly(ADP-ribose) polymerase inhibitor in murine models of heart failure. Int J Mol Med. (2006) 17:369–75. 10.3892/ijmm.17.2.369 16391839 PMC2245862

[62] Ganatra S Nohria A Shah S Groarke JD Sharma A Venesy D Upfront dexrazoxane for the reduction of anthracycline-induced cardiotoxicity in adults with preexisting cardiomyopathy and cancer: a consecutive case series. Cardio-Oncol. (2019) 5:1 10.1186/s40959-019-0036-7 PMC7048095 32154008

[63] Swain SM Whaley FS Gerber MC Ewer MS Bianchine JR Gams RA. Delayed administration of dexrazoxane provides cardioprotection for patients with advanced breast cancer treated with doxorubicin-containing therapy. J Clin Oncol. (1997) 15:1333–40. 10.1200/JCO.1997.15.4.1333 9193324

[64] Swain SM Whaley FS Gerber MC Weisberg S York M Spicer D Cardioprotection with dexrazoxane for doxorubicin-containing therapy in advanced breast cancer. J Clin Oncol. (1997) 15:1318–32. 10.1200/JCO.1997.15.4.1318 9193323

[65] Speyer JL Green MD Zeleniuch-Jacquotte A Wernz JC Rey M Sanger J. ICRF-187 permits longer treatment with doxorubicin in women with breast cancer. J Clin Oncol. (1992) 10:117–27. 10.1200/JCO.1992.10.1.117 1727913

[66] Marty M Espie M Llombart A Monnier A Rapoport BL Stahalova V. Multicenter randomized phase III study of the cardioprotective effect of dexrazoxane (Cardioxane) in advanced/metastatic breast cancer patients treated with anthracycline-based chemotherapy. Ann Oncol. (2006) 17:614–22. 10.1093/annonc/mdj134 16423847

[67] Lipshultz SE Anderson LM Miller TL Gerschenson M Stevenson KE Neuberg DS. Impaired mitochondrial function is abrogated by dexrazoxane in doxorubicin-treated childhood acute lymphoblastic leukemia survivors. Cancer. (2016) 122:946–53. 10.1002/cncr.29872 26762648 PMC4777628

[68] Reichardt P Tabone MD Mora J Morland B Jones RL. Risk-benefit of dexrazoxane for preventing anthracycline-related cardiotoxicity: re-evaluating the European labeling. Future Oncol. (2018) 14:2663–76. 10.2217/fon-2018-0210 29747541

[69] Asselin BL Devidas M Chen L Franco VI Pullen J Borowitz MJ. Cardioprotection and safety of dexrazoxane in patients treated for newly diagnosed T-Cell acute lymphoblastic leukemia or advanced-stage lymphoblastic non-hodgkin lymphoma: a report of the children's oncology group randomized trial pediatric oncology group 9404. J Clin Oncol. (2016) 34:854–62. 10.1200/JCO.2015.60.8851 26700126 PMC4872007

[70] Lipshultz SE Rifai N Dalton VM Levy DE Silverman LB Lipsitz SR. The effect of dexrazoxane on myocardial injury in doxorubicin-treated children with acute lymphoblastic leukemia. N Engl J Med. (2004) 351:145–53. 10.1056/NEJMoa035153 15247354

[71] Seif AE Walker DM Li Y Huang YS Kavcic M Torp K. Dexrazoxane exposure and risk of secondary acute myeloid leukemia in pediatric oncology patients. Pediatr Blood Cancer. (2015) 62:704–9. 10.1002/pbc.25043 24668949 PMC4177031

[72] Rodriguez-Iturbe B Sepassi L Quiroz Y Ni Z Wallace DC Vaziri ND. Association of mitochondrial SOD deficiency with salt-sensitive hypertension and accelerated renal senescence. J Appl Physiol. (1985) 102:255–60. 10.1152/japplphysiol.00513.2006 17023572

[73] Zhan L Li R Sun Y Dou M Yang W He S. Effect of mito-TEMPO, a mitochondria-targeted antioxidant, in rats with neuropathic pain. Neuroreport. (2018) 29:1275–81. 10.1097/WNR.0000000000001105 30052549

[74] Rocha VC Franca LS de Araujo CF Ng AM de Andrade CM Andrade AC. Protective effects of mito-TEMPO against doxorubicin cardiotoxicity in mice. Cancer Chemother Pharmacol. (2016) 77:659–2. 10.1007/s00280-015-2949-7 26712129

[75] Monti E Cova D Guido E Morelli R Oliva C. Protective effect of the nitroxide tempol against the cardiotoxicity of adriamycin. Free Radic Biol Med. (1996) 21:463–70. 10.1016/0891-5849(96)00124-4 8886796

[76] Dey S deMazumder D Sidor A Foster DB O'Rourke B. Mitochondrial ROS drive sudden cardiac death and chronic proteome remodeling in heart failure. Circ Res. (2018) 123:356–71. 10.1161/CIRCRESAHA.118.312708 29898892 PMC6057154

[77] Dickey JS Gonzalez Y Aryal B Mog S Nakamura AJ Redon CE. Mito-tempol and dexrazoxane exhibit cardioprotective and chemotherapeutic effects through specific protein oxidation and autophagy in a syngeneic breast tumor preclinical model. PLoS ONE. (2013) 8:e70575. 10.1371/journal.pone.0070575 23940596 PMC3734284

[78] Szeto HH Birk AV. Serendipity and the discovery of novel compounds that restore mitochondrial plasticity. Clin Pharmacol Ther. (2014) 96:672–83. 10.1038/clpt.2014.174 25188726 PMC4267688

[79] Dai DF Chen T Szeto H Nieves-Cintron M Kutyavin V Santana LF. Mitochondrial targeted antioxidant peptide ameliorates hypertensive cardiomyopathy. J Am Coll Cardiol. (2011) 58:73–82. 10.1016/j.jacc.2010.12.044 21620606 PMC3742010

[80] Szeto HH Liu S Soong Y Wu D Darrah SF Cheng FY. Mitochondria-targeted peptide accelerates ATP recovery and reduces ischemic kidney injury. J Am Soc Nephrol. (2011) 22:1041–52. 10.1681/ASN.2010080808 21546574 PMC3103724

[81] Birk AV Chao WM Bracken C Warren JD Szeto HH. Targeting mitochondrial cardiolipin and the cytochrome c/cardiolipin complex to promote electron transport and optimize mitochondrial ATP synthesis. Br J Pharmacol. (2014) 171:2017–28. 10.1111/bph.12468 24134698 PMC3976619

[82] Szeto HH. First-in-class cardiolipin-protective compound as a therapeutic agent to restore mitochondrial bioenergetics. Br J Pharmacol. (2014) 171:2029–50. 10.1111/bph.12461 24117165 PMC3976620

[83] Cho J Won K Wu D Soong Y Liu S Szeto HH. Potent mitochondria-targeted peptides reduce myocardial infarction in rats. Coron Artery Dis. (2007) 18:215–20. 10.1097/01.mca.0000236285.71683.b6 17429296

[84] Dai W Shi J Gupta RC Sabbah HN Hale SL Kloner RA. Bendavia, a mitochondria-targeting peptide, improves postinfarction cardiac function, prevents adverse left ventricular remodeling, and restores mitochondria-related gene expression in rats. J Cardiovasc Pharmacol. (2014) 64:543–53. 10.1097/FJC.0000000000000155 25165999

[85] Wu D Soong Y Zhao GM Szeto HH. A highly potent peptide analgesic that protects against ischemia-reperfusion-induced myocardial stunning. Am J Physiol Heart Circ Physiol. (2002) 283:H783–91. 10.1152/ajpheart.00193.2002 12124228

[86] Sabbah HN Gupta RC Kohli S Wang M Hachem S Zhang K. Chronic therapy with elamipretide (MTP-131), a novel mitochondria-targeting peptide, improves left ventricular and mitochondrial function in dogs with advanced heart failure. Circ Heart Fail. (2016) 9:e002206. 10.1161/CIRCHEARTFAILURE.115.002206 26839394 PMC4743543

[87] Gupta RC Sing-Gupta V Sabbah HN Bendavia (Elamipretide) restores phosphorylation of cardiac myosin binding protein C on serine 282 and improves left ventricular diastolic function in dogs with heart failure. J Am Coll Cardiol. (2016) 67:1443 10.1016/S0735-1097(16)31444-9

[88] Daubert MA Yow E Dunn G Marchev S Barnhart H Douglas PS. Novel mitochondria-targeting peptide in heart failure treatment: a randomized, placebo-controlled trial of elamipretide. Circ Heart Fail. (2017) 10:e004389. 10.1161/CIRCHEARTFAILURE.117.004389 29217757

[89] Schiattarella GG Hill JA. Therapeutic targeting of autophagy in cardiovascular disease. J Mol Cell Cardiol. (2016) 95:86–93. 10.1016/j.yjmcc.2015.11.019 26602750 PMC4871782

[90] Sciarretta S Yee D Nagarajan N Bianchi F Saito T Valenti V. Trehalose-induced activation of autophagy improves cardiac remodeling after myocardial infarction. J Am Coll Cardiol. (2018) 71:1999–2010. 10.1016/j.jacc.2018.02.066 29724354 PMC6347412

[91] Sishi BJ Loos B van Rooyen J Engelbrecht AM. Autophagy upregulation promotes survival and attenuates doxorubicin-induced cardiotoxicity. Biochem Pharmacol. (2013) 85:124–34. 10.1016/j.bcp.2012.10.005 23107818

[92] Chude CI Amaravadi RK. Targeting autophagy in cancer: update on clinical trials and novel inhibitors. Int J Mol Sci. (2017) 18:1279. 10.3390/ijms18061279 28621712 PMC5486101

[93] Liu Q Docherty JC Rendell JC Clanachan AS Lopaschuk GD. High levels of fatty acids delay the recovery of intracellular pH and cardiac efficiency in post-ischemic hearts by inhibiting glucose oxidation. J Am Coll Cardiol. (2002) 39:718–25. 10.1016/S0735-1097(01)01803-4 11849874

[94] Buchanan J Mazumder PK Hu P Chakrabarti G Roberts MW Yun UJ. Reduced cardiac efficiency and altered substrate metabolism precedes the onset of hyperglycemia and contractile dysfunction in two mouse models of insulin resistance and obesity. Endocrinology. (2005) 146:5341–9. 10.1210/en.2005-0938 16141388

[95] Lopaschuk GD Ussher JR Folmes CD Jaswal JS Stanley WC. Myocardial fatty acid metabolism in health and disease. Physiol Rev. (2010) 90:207–58. 10.1152/physrev.00015.2009 20086077

[96] Stanley WC Recchia FA Lopaschuk GD. Myocardial substrate metabolism in the normal and failing heart. Physiol Rev. (2005) 85:1093–129. 10.1152/physrev.00006.2004 15987803

[97] Dezsi CA. Trimetazidine in practice: review of the clinical and experimental evidence. Am J Ther. (2016) 23:e871–9. 10.1097/MJT.0000000000000180 25756467 PMC4856171

[98] Lopatin YM Rosano GM Fragasso G Lopaschuk GD Seferovic PM Gowdak LH. Rationale and benefits of trimetazidine by acting on cardiac metabolism in heart failure. Int J Cardiol. (2016) 203:909–15. 10.1016/j.ijcard.2015.11.060 26618252

[99] Stanley WC Marzilli M. Metabolic therapy in the treatment of ischaemic heart disease: the pharmacology of trimetazidine. Fundam Clin Pharmacol. (2003) 17:133–45. 10.1046/j.1472-8206.2003.00154.x 12667223

[100] Lionetti V Linke A Chandler MP Young ME Penn MS Gupte S. Carnitine palmitoyl transferase-I inhibition prevents ventricular remodeling and delays decompensation in pacing-induced heart failure. Cardiovasc Res. (2005) 66:454–61. 10.1016/j.cardiores.2005.02.004 15914110

[101] Ma Y Temkin SM Hawkridge AM Guo C Wang W Wang XY. Fatty acid oxidation: an emerging facet of metabolic transformation in cancer. Cancer Lett. (2018) 435:92–100. 10.1016/j.canlet.2018.08.006 30102953 PMC6240910

[102] Tang WH. Metabolic approach in heart failure: rethinking how we translate from theory to clinical practice. J Am Coll Cardiol. (2006) 48:999–1000. 10.1016/j.jacc.2006.06.024 16949493

[103] Zhang L Lu Y Jiang H Sun A Zou Y Ge J. Additional use of trimetazidine in patients with chronic heart failure: a meta-analysis. J Am Coll Cardiol. (2012) 59:913–22. 10.1016/j.jacc.2011.11.027 22381427

[104] Steggall A Mordi IR Lang CC. Targeting metabolic modulation and mitochondrial dysfunction in the treatment of heart failure. Diseases. (2017) 5:14. 10.3390/diseases5020014 28933367 PMC5547981

[105] Zhou X Chen J. Is treatment with trimetazidine beneficial in patients with chronic heart failure? PLoS ONE. (2014) 9:e94660. 10.1371/journal.pone.0094660 24797235 PMC4010408

[106] Gao D Ning N Niu X Hao G Meng Z. Trimetazidine: a meta-analysis of randomised controlled trials in heart failure. Heart. (2011) 97:278–86. 10.1136/hrt.2010.208751 21134903

[107] Jaswal JS Keung W Wang W Ussher JR Lopaschuk GD. Targeting fatty acid and carbohydrate oxidation–a novel therapeutic intervention in the ischemic and failing heart. Biochim Biophys Acta. (2011) 1813:1333–50. 10.1016/j.bbamcr.2011.01.015 21256164

[108] Morrow DA Scirica BM Sabatine MS de Lemos JA Murphy SA Jarolim P. B-type natriuretic peptide and the effect of ranolazine in patients with non-ST-segment elevation acute coronary syndromes: observations from the MERLIN-TIMI 36 (Metabolic efficiency with ranolazine for less ischemia in Non-ST elevation acute coronary-thrombolysis in myocardial infarction 36) trial. J Am Coll Cardiol. (2010) 55:1189–96. 10.1016/j.jacc.2009.09.068 20298924

[109] Hawwa N Menon V. Ranolazine: clinical applications and therapeutic basis. Am J Cardiovasc Drugs. (2013) 13:5–16. 10.1007/s40256-012-0003-2 23335347

[110] Undrovinas AI Belardinelli L Undrovinas NA Sabbah HN. Ranolazine improves abnormal repolarization and contraction in left ventricular myocytes of dogs with heart failure by inhibiting late sodium current. J Cardiovasc Electrophysiol. (2006) 17(Suppl. 1):S169–77. 10.1111/j.1540-8167.2006.00401.x 16686675 PMC1482456

[111] Sabbah HN Chandler MP Mishima T Suzuki G Chaudhry P Nass O. Ranolazine, a partial fatty acid oxidation (pFOX) inhibitor, improves left ventricular function in dogs with chronic heart failure. J Card Fail. (2002) 8:416–22. 10.1054/jcaf.2002.129232 12528095

[112] Rastogi S Sharov VG Mishra S Gupta RC Blackburn B Belardinelli L. Ranolazine combined with enalapril or metoprolol prevents progressive LV dysfunction and remodeling in dogs with moderate heart failure. Am J Physiol Heart Circ Physiol. (2008) 295:H2149–55. 10.1152/ajpheart.00728.2008 18820026 PMC2614581

[113] Maier LS Layug B Karwatowska-Prokopczuk E Belardinelli L Lee S Sander J. RAnoLazIne for the treatment of diastolic heart failure in patients with preserved ejection fraction: the RALI-DHF proof-of-concept study. JACC Heart Fail. (2013) 1:115–22. 10.1016/j.jchf.2012.12.002 24621836

[114] Ashrafian H Horowitz JD Frenneaux MP. Perhexiline. Cardiovasc Drug Rev. (2007) 25:76–97. 10.1111/j.1527-3466.2007.00006.x 17445089

[115] Kennedy JA Unger SA Horowitz JD. Inhibition of carnitine palmitoyltransferase-1 in rat heart and liver by perhexiline and amiodarone. Biochem Pharmacol. (1996) 52:273–80. 10.1016/0006-2952(96)00204-3 8694852

[116] Lee L Campbell R Scheuermann-Freestone M Taylor R Gunaruwan P Williams L. Metabolic modulation with perhexiline in chronic heart failure: a randomized, controlled trial of short-term use of a novel treatment. Circulation. (2005) 112:3280–8. 10.1161/CIRCULATIONAHA.105.551457 16301359

[117] Leask A. Getting to the heart of the matter: new insights into cardiac fibrosis. Circ Res. (2015) 116:1269–76. 10.1161/CIRCRESAHA.116.305381 25814687

[118] Cappetta D Esposito G Piegari E Russo R Ciuffreda LP Rivellino A. SIRT1 activation attenuates diastolic dysfunction by reducing cardiac fibrosis in a model of anthracycline cardiomyopathy. Int J Cardiol. (2016) 205:99–110. 10.1016/j.ijcard.2015.12.008 26730840

[119] Zhan H Aizawa K Sun J Tomida S Otsu K Conway SJ. Ataxia telangiectasia mutated in cardiac fibroblasts regulates doxorubicin-induced cardiotoxicity. Cardiovasc Res. (2016) 110:85–95. 10.1093/cvr/cvw032 26862121 PMC4798048

[120] Wolf MB Baynes JW. The anti-cancer drug, doxorubicin, causes oxidant stress-induced endothelial dysfunction. Biochim Biophys Acta. (2006) 1760:267–71. 10.1016/j.bbagen.2005.10.012 16337743

[121] Chatterjee K Zhang J Honbo N Karliner JS. Doxorubicin cardiomyopathy. Cardiology. (2010) 115:155–62. 10.1159/000265166 20016174 PMC2848530

[122] Magdy T Burridge PW. The future role of pharmacogenomics in anticancer agent-induced cardiovascular toxicity. Pharmacogenomics. (2018) 19:79–82. 10.2217/pgs-2017-0177 29199515

[123] Pinheiro EA Fetterman KA Burridge PW. HiPSCs in cardio-oncology: deciphering the genomics. Cardiovasc Res. (2019) 115:935–48. 10.1093/cvr/cvz018 30689737 PMC6452310

[124] Tripaydonis A Conyers R Elliott DA. Pediatric anthracycline-induced cardiotoxicity: mechanisms, pharmacogenomics, and pluripotent stem-cell modeling. Clin Pharmacol Ther. (2019) 105:614–24. 10.1002/cpt.1311 30460992 PMC6590110

[125] Knowles DA Burrows CK Blischak JD Patterson KM Serie DJ Norton N. Determining the genetic basis of anthracycline-cardiotoxicity by molecular response QTL mapping in induced cardiomyocytes. Elife. (2018) 7:e33480. 10.7554/eLife.33480 29737278 PMC6010343

[126] Burridge PW Li YF Matsa E Wu H Ong SG Sharma A. Human induced pluripotent stem cell-derived cardiomyocytes recapitulate the predilection of breast cancer patients to doxorubicin-induced cardiotoxicity. Nat Med. (2016) 22:547–56. 10.1038/nm.4087 27089514 PMC5086256

